# EpCAM-CD24+ circulating cells associated with poor prognosis in breast cancer patients

**DOI:** 10.1038/s41598-024-61516-2

**Published:** 2024-05-28

**Authors:** V. M. Perelmuter, E. S. Grigoryeva, O. E. Savelieva, V. V. Alifanov, E. S. Andruhova, M. V. Zavyalova, O. D. Bragina, E. Yu. Garbukov, M. E. Menyailo, A. A. Khozyainova, E. V. Denisov, N. V. Cherdyntseva, L. A. Tashireva

**Affiliations:** 1 The Department of General and Molecular Pathology, Cancer Research Institute, Tomsk National Research Medical Center, Russian Academy of Sciences, Tomsk, Russia; 2 The Laboratory of Molecular Therapy of Cancer, Cancer Research Institute, Tomsk National Research Medical Center, Russian Academy of Sciences, Tomsk, Russia; 3 The Laboratory of Molecular Oncology and Immunology, Cancer Research Institute, Tomsk National Research Medical Center, Russian Academy of Sciences, Tomsk, Russia; 4 The Department of General Oncology, Cancer Research Institute, Tomsk National Research Medical Center, Russian Academy of Sciences, Tomsk, Russia; 5 The Laboratory of Cancer Progression Biology, Cancer Research Institute, Tomsk National Research Medical Center, Russian Academy of Sciences, Tomsk, Russia

**Keywords:** Cancer, Breast cancer, Tumour heterogeneity

## Abstract

Following the discovery of circulating tumor cells (CTCs) in the peripheral blood of cancer patients, CTCs were initially postulated to hold promise as a valuable prognostic tool through liquid biopsy. However, a decade and a half of accumulated data have revealed significant complexities in the investigation of CTCs. A challenging aspect lies in the reduced expression or complete loss of key epithelial markers during the epithelial-mesenchymal transition (EMT). This likely hampers the identification of a pathogenetically significant subset of CTCs. Nevertheless, there is a growing body of evidence regarding the prognostic value of such molecules as CD24 expressing in the primary breast tumor. Herewith, the exact relevance of CD24 expression on CTCs remains unclear. We used two epithelial markers (EpCAM and cytokeratin 7/8) to assess the count of CTCs in 57 breast cancer patients, both with (M0^mts^) and without metastasis (M0) during the follow-up period, as well as in M1 breast cancer patients. However, the investigation of these epithelial markers proved ineffective in identifying cell population expressing different combinations of EpCAM and cytokeratin 7/8 with prognostic significance for breast cancer metastases. Surprisingly, we found CD24+ circulating cells (CCs) in peripheral blood of breast cancer patients which have no epithelial markers (EpCAM and cytokeratin 7/8) but was strongly associated with distant metastasis. Namely, the count of CD45-EpCAM-CK7/8-CD24+ N-cadherin—CCs was elevated in both groups of patients, those with existing metastasis and those who developed metastases during the follow-up period. Simultaneously, an elevation in these cell counts beyond the established threshold of 218.3 cells per 1 mL of blood in patients prior to any treatment predicted a 12-fold risk of metastases, along with a threefold decrease in distant metastasis-free survival over a 90-month follow-up period. The origin of CD45-EpCAM-CK7/8-CD24+ N-cadherin—CCs remains unclear. In our opinion their existence can be explained by two most probable hypotheses. These cells could exhibit a terminal EMT phenotype, or it might be immature cells originating from the bone marrow. Nonetheless, if this hypothesis holds true, it's worth noting that the mentioned CCs do not align with any of the recognized stages of monocyte or neutrophil maturation, primarily due to the presence of CD45 expression in the myeloid cells. The results suggest the presence in the peripheral blood of patients with metastasis (both during the follow-up period and prior to inclusion in the study) of a cell population with a currently unspecified origin, possibly arising from both myeloid and tumor sources, as confirmed by the presence of aneuploidy.

## Introduction

Breast cancer ranks among the prevalent malignancies afflicting women^1^, and despite substantial progress in therapeutic protocols, screening, and vigilant monitoring, the risk of disease recurrence persists for years following diagnosis^2^. The search of informative markers that can accurately predict the progression of breast cancer remains a pressing and contemporary challenge.

After discovering the circulating tumor cells (CTCs) in peripheral blood of cancer patients it was assumed that they can serve as a valuable instrument for prognosis of clinical course through liquid biopsy. However, the data amassed during the preceding 15 years have elucidated several notable complexities in the investigation of CTCs. Many isolation and detection methods are used for CTC research, the sensitivity and specificity of which vary considerably. Thus, the lack of standardization of methods leads to inconsistency of results of different research groups and impossibility to compare the obtained data. Another significant problem is the lack of universal CTC markers. It turned out that there are cancer types that express low or no EpCAM^3,4^, which means that the use of EpCAM-based commercial platforms such as CELLSEARCH is extremely limited.

Numerous data demonstrates the role of CD24, an anchored cell surface glycoprotein, as a regulator of cell migration, invasion and proliferation^5,6^. In breast cancer, the expression of CD24 was significantly higher in invasive cancer than in normal tissues^7^. Expression of CD24 on the cell surface and in the cytoplasm was associated with tumor size, histologic grade, lymph node positivity, and poor prognosis^8^. Only one study of CD24 expression on CTCs was carried out and revealed that CD24+ CTCs, especially with EMT features, may be one of the prognostic indicators for patients with early and intermediate stage breast cancer^9^. The authors used CanPatrol CTC enrichment technology, so the analysis included CTCs with epithelial (EpCAM, CK8/18/19) and mesenchymal (vimentin, twist) features. In our study, we examined the entire pool of CD45− cells depending on CD24 expression. We assessed the relationships between the count of CTCs and CCs and the occurrence and presence of distant metastases. The aim of this study was to clarify the prognostic significance of EpCAM-CD24+ CCs in patients with breast cancer.

## Materials and methods

### Patients

The prospective study included 57 patients with invasive breast carcinoma of no special type (IC NST) T1-4N0-3M0-1, admitted for treatment to Cancer Research Institute, Tomsk National Research Medical Center. The procedure of the study was approved by the Local Committee for Medical Ethics of the institute (17 June 2016, the approval No. 8), and informed consent was obtained from all patients prior to analysis. Venous ethylenediaminetetraacetic acid (EDTA) blood samples were taken before surgery and neoadjuvant chemotherapy. The study was performed in accordance with the principles outlined in the Declaration of Helsinki. Patients were treated according to ESMO Clinical Practice Guidelines^10^. Patients were informed about the purpose and possible risks of the study, and all gave their informed consent.

### Blood specimen collection and processing for CTCs immunophenotyping

The sample processing and immunophenotyping was performed as described in our previous study^11^. Blood samples were collected to EDTA pre-coated 9 mL tubes, then incubated at 37 °C for 1.5 h. White blood cells were aspirated from thin white layer between plasma and red blood cells after their sedimentation. Obtained cell concentrate washed in 2 mL Cell Wash buffer (BD Biosciences, USA) by centrifugation at 800× g for 15 min and resuspended in 150 μl of sterile PBS.

### Flow cytometry

Surface markers (CD45, EpCAM (CD326), CD44, CD24, CD133, N-cadherin (CD325)), were stained by first step, intracellular staining was performed on second step. Monoclonal antibodies were added and incubated at RT for 20 min: APC-Cy7-anti-CD45 (clone HI30, IgG1, Sony Biotechnology, USA), BV 650-anti-EpCAM (clone 9C4, IgG2b, Sony Biotechnology, USA), PE-Cy7-anti-N-cadherin (clone 8C11, IgG1, Sony Biotechnology, USA), PerCP-Cy5.5-anti-CD24 (clone ML5, IgG2a, Sony Biotechnology, USA), BV 510-anti-CD44 (clone G44-26, IgG2b, BD Horizont, USA). The unstained control and antibody quality control was performed. The appropriate isotype antibodies were added to the isotype control sample at the same concentration. After incubation, red blood cells were lysed by 250 μL OptiLyse C buffer (Beckman Coulter, France) at RT for 10 min in dark and washed in 1 mL Cell Wash buffer (BD Biosciences, USA) at 800× g for 6 min.

For intracellular staining cells were permeabilized by 250 μL BD Cytofix/Cytoperm (BD Biosciences, USA) at 4 °C for 30 min in the dark and washed twice in 1 mL BD Perm/Wash buffer (BD Biosciences, USA) at 800× g for 6 min. After samples were diluted in 50 μL BD Perm/Wash buffer (BD Biosciences, USA) and incubated at 4 °C for 10 min in dark with 5 μL of Fc Receptor Blocking Solution (Human TruStain FcX, Sony Biotechnology, USA). Next, monoclonal antibodies anti-CK7/8-PE (clone CAM5.2, BD Biosciences, USA) were added and incubated at 4 °C for 20 min. The appropriate isotype control antibodies at the same concentration were added to the control sample. After incubation, samples were washed in 1 mL Cell Wash buffer (BD Biosciences, USA) at 800× g for 6 min. After samples were diluted in 100 μL Stain buffer (Sony Biotechnology, USA). Compensation beads (VersaComp Antibody Capture Bead kit, Beckman Coulter, USA) were used for compensation control. The immunofluorescence was analyzed on the Novocyte 3000 (ACEA Biosciences, USA).

**Figure 1:**
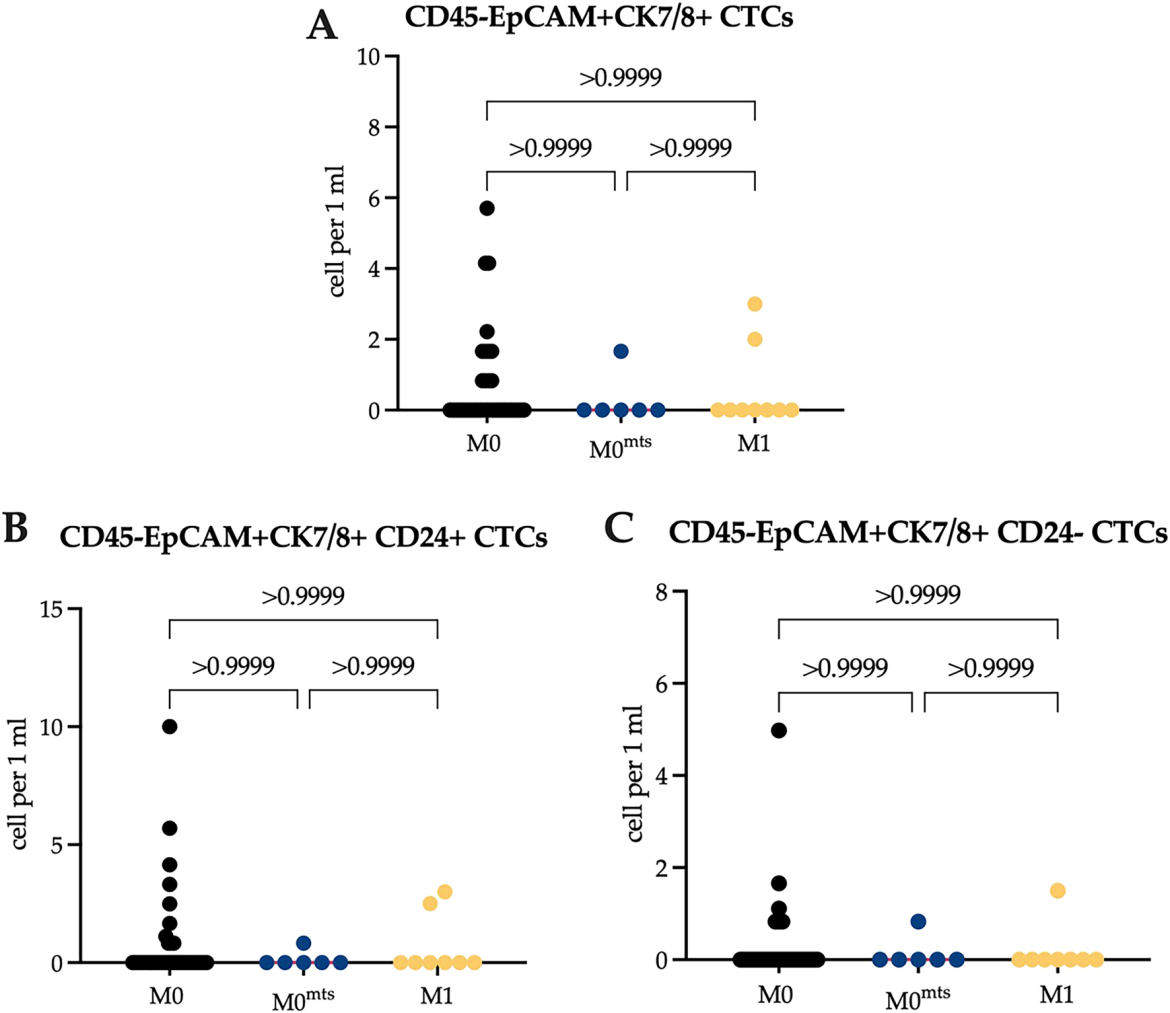
The number of EpCAM+ CK7/8+ CTCs in relation to CD24 expression in studied groups of patients. (**A**) The total number of EpCAM+ CK7/8+ CTCs in patients with no metastasis during follow-up period (M0), with distant metastasis during follow-up period (M0^mts^) and in metastatic breast cancer patients (M1). (**B**) The number of EpCAM+ CK7/8+ CD24+ CTCs in M0, M0^mts^ and M1 breast cancer patients. (**C**) The number of EpCAM+ CK7/8+ CD24− CTCs in M0, M0^mts^ and M1 breast cancer patients. P-values represented for all comparison groups.

Gating strategy was as follow: using forward (FSC) and side scatter (SSC) gates debris was discriminate, doublets was also discriminate by plotting FSC area vs FSC height. Follow analysis included only CD45-negative cells which were gated using quadrant-based scheme by EpCAM and CK7/8 expression to distinguish the subsets: EpCAM+ CK7/8+, EpCAM+ CK7/8-, EpCAM-CK7/8+ and EpCAM-CK7/8-. The expression of CD44, CD24, and N-cadherin was evaluated in each population. Representative example of the supervised gating and analysis strategy of flow cytometry data provided in Supplementary (Fig. 1).

### DNA ploidity analysis

DNA ploidy were analyzed on scRNA-seq data generated in our previous study are available via BioProject under the accession number PRJNA776403 using CopyKAT instrument^12^. Raw gene expression matrices from patients with PTPRC-/EPCAM-/KRT7-/KRT8−/CD24+ /CDH2− phenotype cells were imported into Seurat v 4.2.1 and the following parameters were used in analysis: the minimal number of genes per chromosome for cell filtering—2, minimal window sizes for segmentation—25 genes per segment, segmentation parameter—0.15. Barcodes belonging to helper T cells were used as a normal cell vector. Helper T cells were identified as cells with no epithelial genes (EPCAM, CDH2, KRT5, KRT7, KRT8, KRT18) and expression level of PTPRC (CD45) and CD4 genes more than 0.

### Statistical analysis

The data was analyzed using the GraphPad Prism 9 (GraphPad Software, San Diego, CA, USA). The Mann–Whitney test was used to compare differences between independent group, for the dependent variables Wilcoxon test was used. Overall and metastasis-free survival was calculated by the Kaplan–Meier method, and differences in survival curves among the groups were evaluated by the log rank test. Metastasis-free survival was assessed with univariate and multivariate Cox regression models and resulted in hazard ratios (HRs). This model adjusts for age, molecular subtype, tumor size, neoadjuvant chemotherapy, lymph node involvement and number of CD24+ CCs. Cox regression analysis was performed to assess the prediction power of the number of CD24+ non-epithelial CCs in metastasis-free survival. Akaike’s information criterion (AIC) was used for prognostic models’ comparison. p < 0.05 was considered statistically significant.

## Results

The full clinicopathological parameters of patients are presented in Tables 1 and 2. Three groups of breast cancer patients were studied: M0—patients without metastases in follow-up period (Table 1); M0^mts^—patients with distant metastases in follow-up period (Table 1); M1—metastatic breast cancer patients (Table 2).

**Table 1:** Clinicopathological characteristics of M0 patients.

Parameter		Frequency, % (n)		P-value
		M0	M0^mts^	
Age	< 35	7.32% (3/41)	14.29% (1/7)	
	35–50	31.71% (13/41)	57.14% (4/7)	
	> 50	60.97% (25/41)	28.57% (2/7)	
Menstrual function	Premenopausal	41.46% (17/41)	28.57% (2/7)	
	Postmenopausal	58.54% (24/41)	71.43% (5/7)	
Tumor size (T)	1	39.02% (16/41)	0.00% (0/7)	
	2	60.98% (25/41)	57.14% (4/7)	
	3	0.0% (0/41)	0.00% (0/7)	
	4	0.0% (0/41)	42.86% (3/7)	p = 0.008
Stage	I	31.71% (13/41)	0.00% (0/7)	
	IIA	51.22% (21/41)	28.57% (2/7)	
	IIB	14.63% (6/41)	14.29% (1/7)	
	IIIA	0.0% (0/41)	0.0% (0/7)	
	IIIB	0.0% (0/41)	28.57% (2/7)	p = 0.02
	IIIC IV	2.44% (1/41)	28.57% (2/7)	p = 0.03
		0.00% (0/41)	0.00% (0/7)	
Molecular subtype	Luminal A	31.71% (13/41)	14.29% (1/7)	
	Luminal B (HER2-)	34.15% (14/41)	42.85% (3/7)	
	Luminal B (HER2+)	17.07% (7/41)	14.29% (1/7)	
	Triple negative	14.63% (6/41)	28.57% (2/7)	
	HER2-enriched	2.44% (1/41)	0.00% (0/7)	
Lymph node metastasis	Yes	75.61% (31/41)	100.00% (7/7)	
	No	24.39% (10/41)	0.00% (0/7)	
Neoadjuvant chemotherapy	Yes	73.81% (31/41)	71.43% (5/7)	
	No	26.19% (10/41)	28.57% (2/7)

**Table 2:** Clinicopathological characteristics of M1 patients.

Parameter		Frequency, % (n)
Age	< 35	0.0% (0/9)
	35–50	0.0% (0/9)
	> 50	100.0% (9/9)
Menstrual function	Premenopausal	0.0% (0/9)
	Postmenopausal	100.0% (9/9)
Tumor size (T)	1	11.11% (1/9)
	2	33.33% (3/9)
	3	0.0% (0/9)
	4	55.56% (5/9)
Stage	I	0.0% (0/9)
	IIA	0.0% (0/9)
	IIB	0.0% (0/9)
	IIIA	0.0% (0/9)
	IIIB	0.0% (0/9)
	IIIC IV	0.0% (0/9)
		100.0% (9/9)
Molecular subtype	Luminal A	0.0% (0/9)
	Luminal B (HER2-)	33.33% (3/9)
	Luminal B (HER2+)	33.33% (3/9)
	Triple negative	33.33% (3/9)
	HER2-enriched	0.0% (0/9)
Lymph node metastasis	Yes	44.44% (4/9)
	No No data	0.0% (0/9) 55.56% (5/9)
Distant metastasis	Yes	100.0% (9/9)
	No	0.0% (0/9)
Neoadjuvant chemotherapy	Yes	0.0% (0/9)
	No	100.0% (9/9)

The number of patients with large tumor size (T4) was higher in group of M0^mts^ patients compared to M0 breast cancer patients (p = 0.008) (Table 1). Patients with stage IIIB and IIIC were most frequently detected in the M0^mts^ group (p = 0.02 and p = 0.03, respectively) (Table 1).

In this study, we used CD45, EpCAM, and CK7/8 to identify circulating tumor cells (CTCs, CD45-EpCAM+ CK7/8+), as well as circulating cancer cells exhibiting monoexpression of epithelial markers—CD45-EpCAM+CK7/8 and CD45-EpCAM-CK7/8+ (Epit-CCs). The evaluation of CD45-EpCAM+CK7/8− and CD45-EpCAM-CK7/8+ subpopulation along with EpCAM+CK7/8+ CTCs allowed us to overcome the limitations associated with the use of CELLSEARCH technology. We also focused on CD45-EpCAM-CK7/8− circulating cells (CCs) that did not have studied epithelial markers, although they could have a tumor origin veiled by terminal stage of EMT.

### The number of CTCs and Epit-CCs in breast cancer patients

Comparison of the total number of EpCAM+CK7/8+ CTCs in M0 and M0^mts^ patients as well as in M1 patients revealed no significant difference (p > 0.05). The median was 0.00 (0.00–0.83), 0.00 (0.00–0.42) and 0.00 (0.00–1.00) cells/mL, respectively (Fig. 1A). The median of EpCAM+CK7/8+ CTCs with CD24-positive expression was 0.00 (0.00–0.83), 0.00 (0.00–0.21) and 0.00 (0.00–0.38) cells/mL, respectively (Fig. 1B); the count of EpCAM+CK7/8+CD24− CTCs was 0.00 (0.00–0.00), 0.00 (0.00–0.21) and 0.00 (0.00–0.00) cells/mL, respectively (Fig. 1C). The number of CD24+ and CD24− CTCs did not significantly differ in patients of the studied groups (p > 0.05).

**Figure 2:**
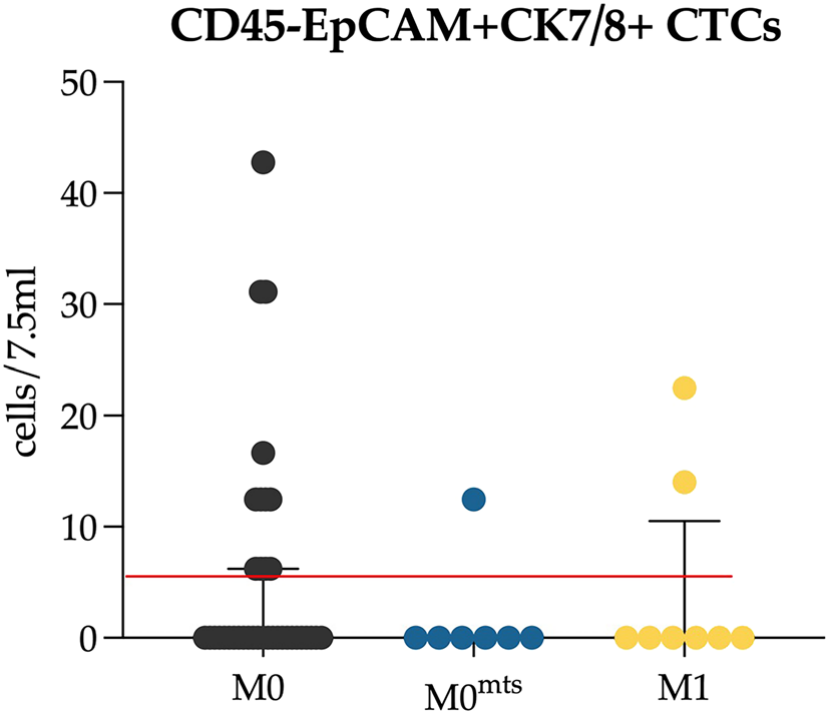
The number of EpCAM+CK7/8+ CTCs which could be potentially detected by the CELLSEARCH system in 7.5 mL peripheral blood in our study. Red line corresponds to cut-off 5 CTCs/ml.

It should be noted that the prognostically significant threshold of 5 cells per 7.5 mL of blood^13–15^ was exceeded in 12 out of 41 patients without metastasis in the follow-up period (M0), and in two metastatic breast cancer patients (Fig. 2). Only one patient belonging to the M0^mts^ group demonstrated enough CTCs to be detected using the CELLSEARCH system. It's worth noting that the only patient cohort suitable for routine CTCs evaluation using CELLSEARCH technology comprises metastatic breast cancer patients. Detection of more than 5 CTCs in 7.5 ml of peripheral blood indicates an unfavorable prognosis for the disease course^16^.

**Figure 3:**
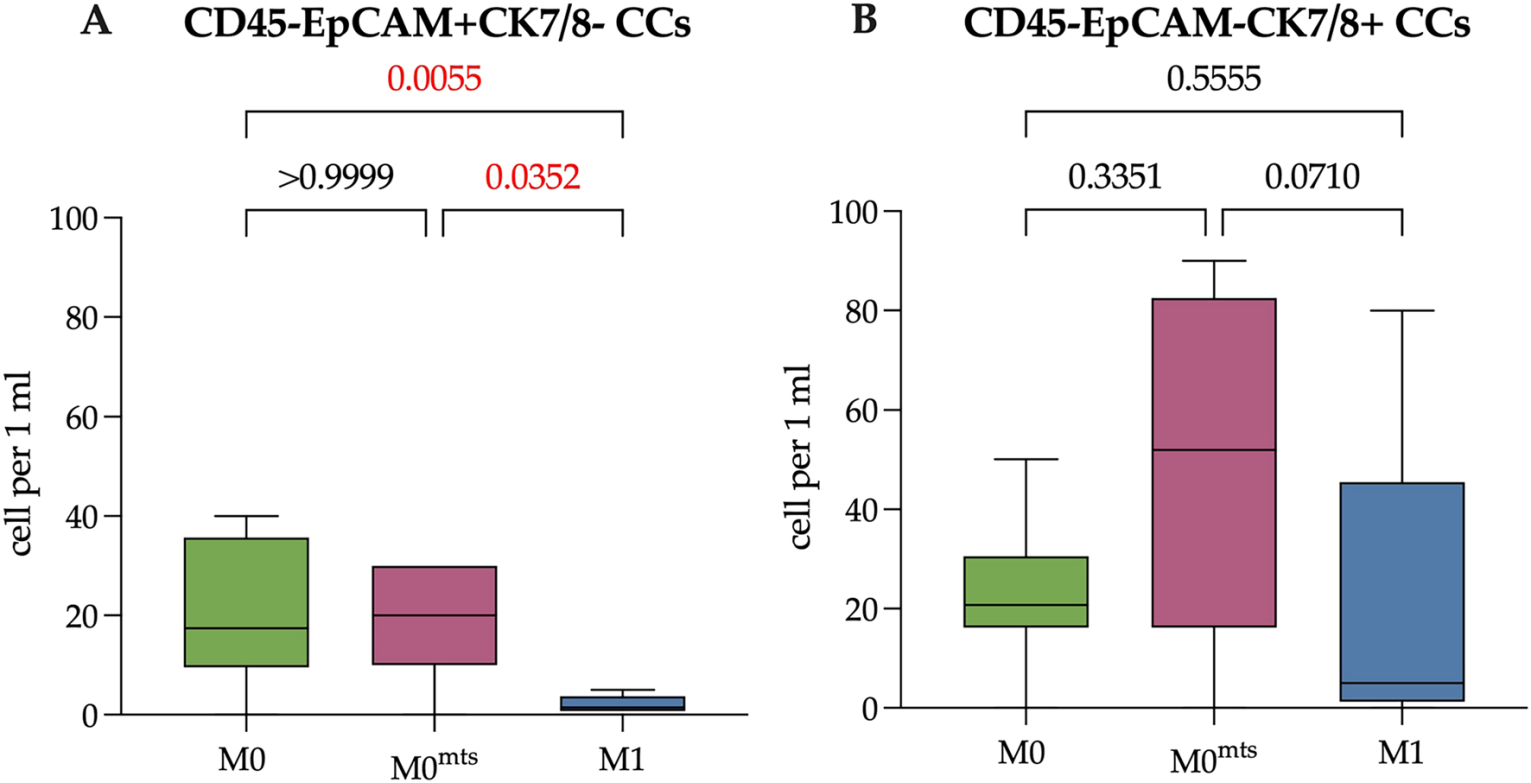
The number of EpCAM+CK7/8− (**A**) and EpCAM-CK7/8+ (**B**) Epit-CCs in patients with no metastasis during follow-up period (M0), with metastasis during follow-up period (M0^mts^) and in metastatic breast cancer patients (M1). P-values represented for all comparison groups.

By using flow cytometry method, we were able to detect EpCAM+CK7/8− and EpCAM-CK7/8+ Epit-CCs that would have been missed when utilizing CELLSEARCH technology (Fig. 3). The number of EpCAM+CK7/8− Epit-CCs in peripheral blood of patients belonging to group M0, M0^mts^ and M1 was 17.43 (9.54–35.69), 20.00 (10.00–30.00) and 1.50 (0.75–3.75) cells/mL (Fig. 3A). Moreover, the number of EpCAM+CK7/8− cells was significantly lower in M1 patients compared to M0 and M0^mts^ patients (p = 0.0055 and p = 0.0352, respectively). The number of EpCAM-CK7/8+ Epit-CCs was as follows: 20.75 (16.23–30.54) in M0 group, 52.00 (16.19–82.50) in M0^mts^ and 5.00 (1.25–45.50) cells/mL in M1 patients (Fig. 3B). The cell count did not significantly differ in the studied groups (p > 0.05).

**Figure 4:**
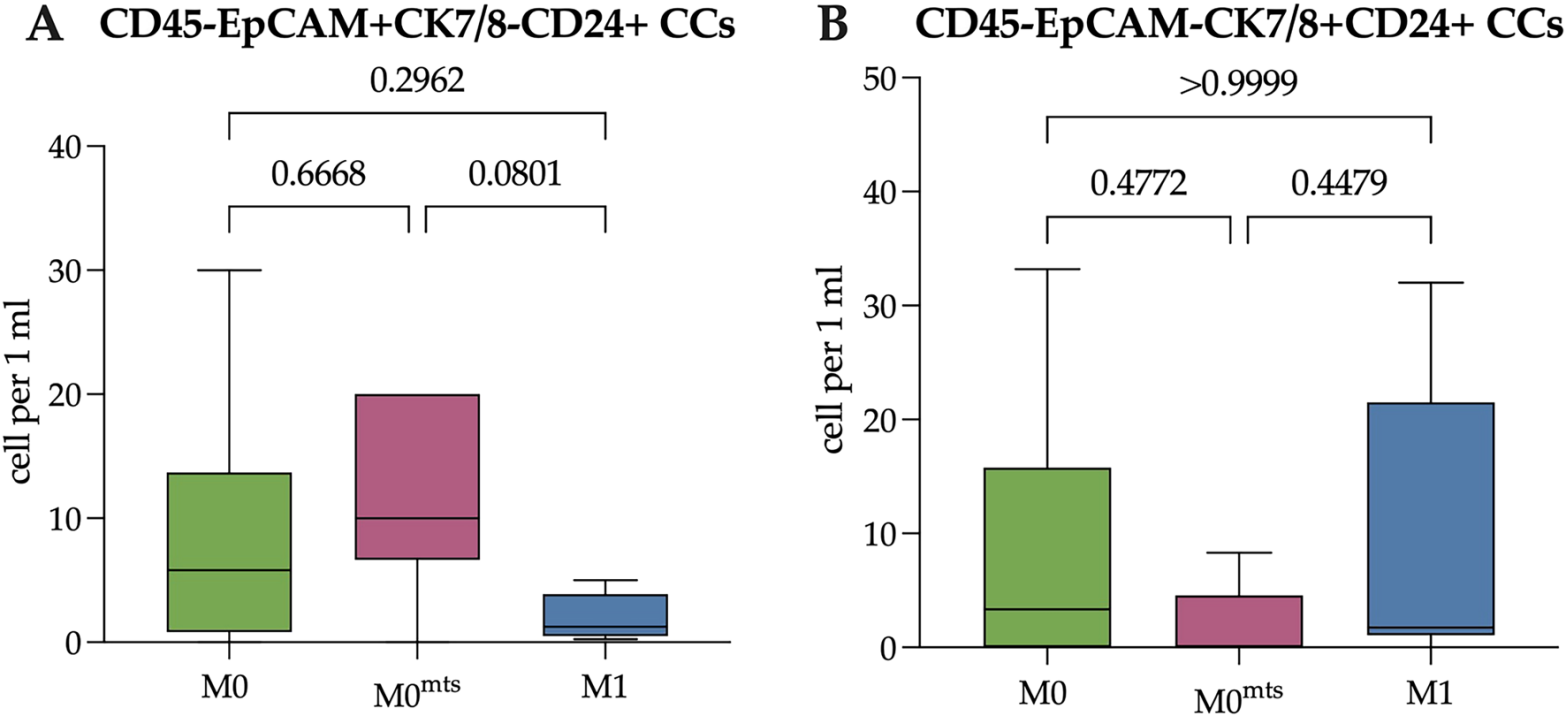
The number of CD24+ Epit-CTCs in patients with no metastasis during follow-up period (M0), with metastasis during follow-up period (M0^mts^) and in metastatic breast cancer patients (M1). (**A**) The number of EpCAM+CK7/8-CD24+ Epit-CTCs in M0, M0^mts^ and M1 breast cancer patients. (**B**) The number of EpCAM-CK7/8+CD24+ Epit-CCs in M0, M0^mts^ and M1 breast cancer patients. P-values represented for all comparison groups.

Next, we evaluated the number of CD24-positive cells among the EpCAM+CK7/8− and EpCAM-CK7/8+ Epit-CCs (Fig. 4).

It was found that there was no difference in the number of CD24-positive EpCAM+CK7/8− and EpCAM-CK7/8+ Epit-CCs, among the studied groups of patients (Fig. 4). The median of EpCAM+ CK7/8-CD24+ Epit-CCs was 5.81 (0.83–13.7), 10.00 (6.64–20.00) and 1.25 (0.50–3.88) cell/mL, respectively (p > 0.05). The number of EpCAM-CK7/8+ CD24+ Epit-CCs in M0 patients was 3.34 (0.00–15.77) cells/mL, (0.00 (0.00–4.56) cells/mL in M0^mts^ patients and 1.75 (1.06–21.50) cells/mL in M1 patients (p > 0.05).

### The number of EpCAM-CK7/8− CCs in breast cancer patients

**Figure 5:**
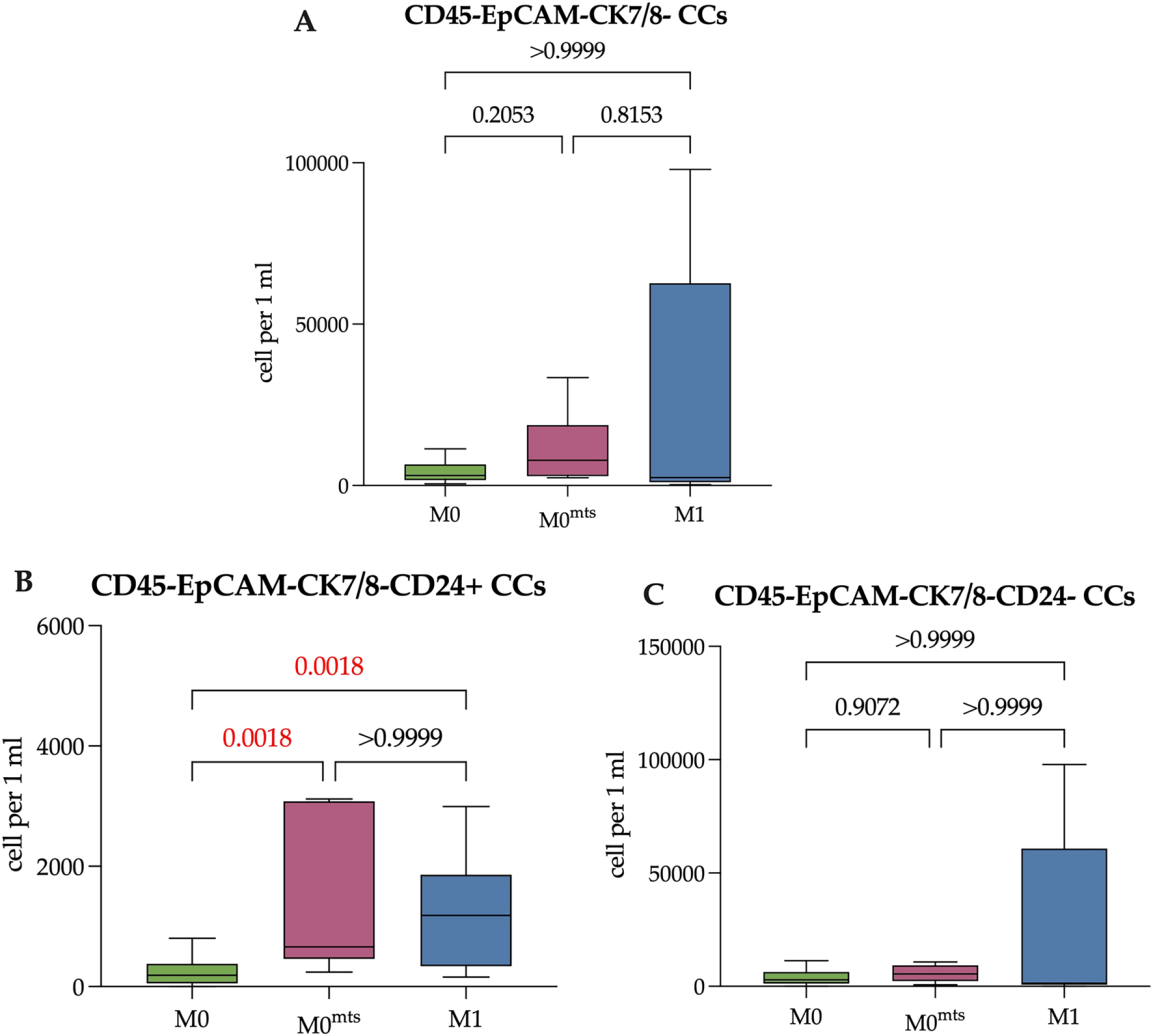
The number of EpCAM-CK7/8- CCs in studied groups of patients. (**A**) The number of EpCAM-CK7/8- CCs in patients with no metastasis during follow-up period (M0), with metastasis during follow-up period (M0^mts^) and in metastatic breast cancer patients (M1). (**B**) The number of EpCAM-CK7/8-CD24+ CCs in M0, M0^mts^ and M1 breast cancer patients. (**C**) The number of EpCAM-CK7/8-CD24- CCs in M0, M0^mts^ and M1 breast cancer patients. P-values represented for all comparison groups.

The number of EpCAM-CK7/8− CCs in breast cancer M0 patients did not differ from those in patients of M0^mts^ and M1 groups, totaling 3174.00 (1707.00–6540.00), 7810.00 (2923.00–18,707.00), and 2513.00 (1023.00–62,666.00) cells/mL, respectively (Fig. 5A) (p > 0.05). The same pattern was observed while comparing the number of EpCAM-CK7/8-CD24− CCs in studied groups (p > 0.05). The median of EpCAM-CK7/8− CD24− CCs in studied groups was as follows: 2820 (1245–6305) cells/mL in M0 patients; 5421.00 (2325.00–9288.00) cells/mL in M0^mts^ patients and 1327.00 (683.30–60,732.00) cells/mL (Fig. 5C).

The number of EpCAM-CK7/8-CD24+ CCs were significantly higher in M0^mts^ and M1 patients (660.00 (460.00–3080.00) and 1185.00 (339.80–1858.00) cells/mL) compared to M0 patients (186.8 (53.86–376.80) cells/mL, p = 0.0018, p = 0.0018, respectively) (Fig. 5B).

**Figure 6:**
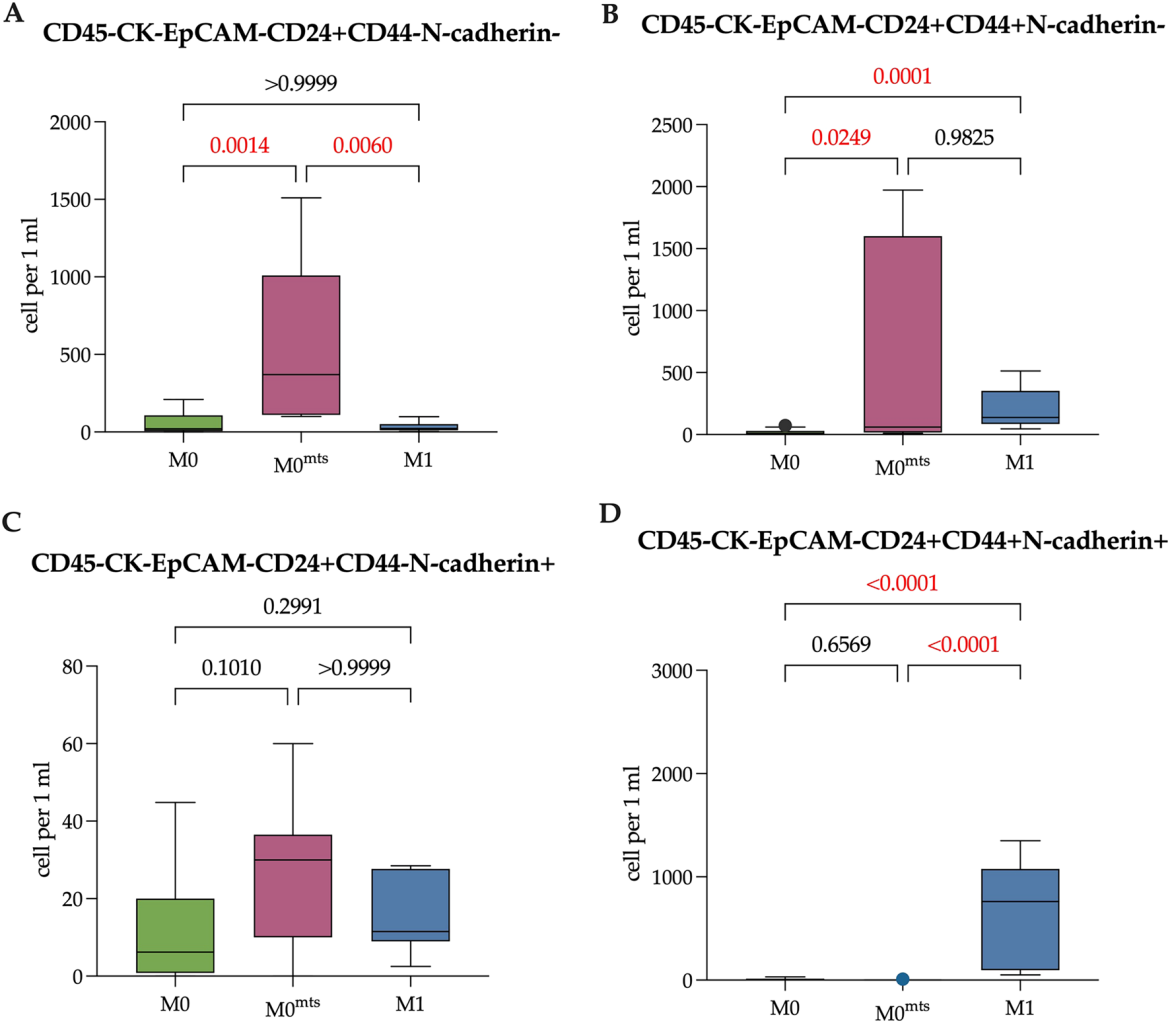
The number of EpCAM-CK7/8-CD24+ CCs in relation to CD44 and N-cadherin expression. (**A**) The number of EpCAM-CK7/8-CD24+CD44-N-cadherin- CCs in patients with no metastasis during follow-up period (M0), with metastasis during follow-up period (M0^mts^) and in metastatic breast cancer patients (M1). (**B**) The number of EpCAM-CK7/8-CD24+CD44+N-cadherin- CCs in M0, M0^mts^ and M1 patients. (**C**) The number of EpCAM-CK7/8-CD24+CD44-N-cadherin+ CCs in M0, M0^mts^ and M1 patients. (**D**) The number of EpCAM-CK7/8-CD24+CD44+N-cadherin+ CCs in M0, M0^mts^ and M1 patients. P-values represented for all comparison groups.

Next, we assessed the expression of CD44 and N-cadherin in EpCAM-CK7/8-CD24+ CCs (Fig. 6). So, the number of N-cadherin- CCs regardless of the CD44 expression in M0^mts^ patients was higher compared to M0 (both in CD44- and CD44+ subpopulations, p = 0.0014 and p = 0.0249, respectively) (Fig. 6A,B). The median of EpCAM-CK7/8-CD24+ CD44-N-cadherin- CCs was 20.75 (5.86–107.50) and 370 (110.00–1009.00) cells/ml in M0 and M0^mts^ patients, while the median of EpCAM-CK7/8-CD24+CD44+N-cadherin- CCs was 10.00 (0.90–30.00) and 60.00 (16.60–1600) cells/ml, respectively.

The number of EpCAM-CK7/8-CD24+CD44-N-cadherin- CCs was also higher in M0^mts^ patients (370 (110.00–1009.00) cells/ml) compared to metastatic breast cancer patients (M1) (22.80 (10.25–51.75) cells/ml (p = 0.0060), while the number of EpCAM-CK7/8-CD24+CD44+N-cadherin- CCs was higher in M1 patients (138.3 (84.94–352.0) cells/ml) compared to patients with no metastasis in follow-up period and counted 10.00 (0.90–30.00) cell/ml and (p = 0.0001) (Fig. 6B).

The number of EpCAM-CK7/8-CD24+CD44+N-cadherin+ CCs was higher in M1 patients compared to M0 and M0^mts^ patients, totaling 760 (97.25–1078.00), 4.85 (0.00–13.28) and 0.00 (0.00–3.75) cells/ml, respectively (p > 0.0001 and p > 0.0001) (Fig. 6D).

To assess the prognostic role of the EpCAM-CK7/8-CD24+ population, since CD44 expression was not relevant, we combined CD44- and CD44+ subpopulations of CCs and further evaluated the differences of EpCAM-CK7/8-CD24+N-cadherin- CCs in M0 and M0^mts^ breast cancer patients.

**Figure 7:**
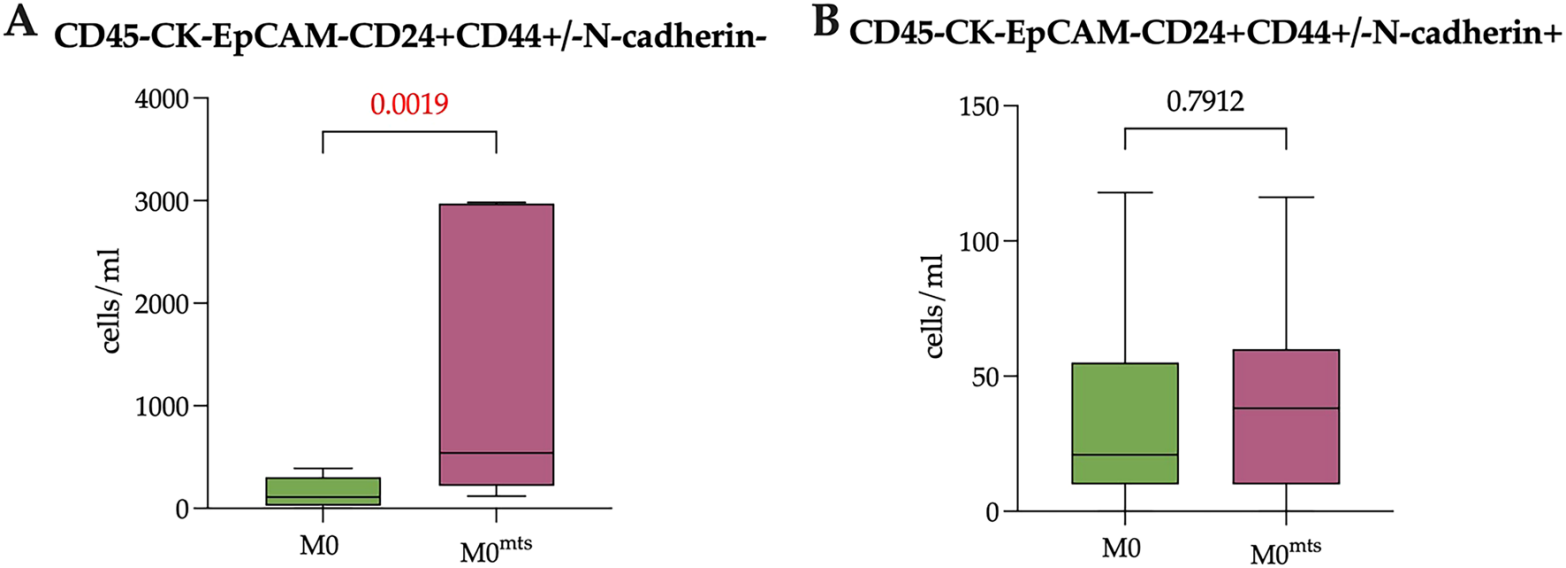
The number of EpCAM-CK7/8-CD24+CD44 ± CCs taking into account N-cadherin expression in patients with no metastasis during follow-up period (M0) and with metastasis during follow-up period (M0^mts^). (**A**) The number of EpCAM-CK7/8-CD24+CD44 ±N-cadherin- CCs in M0 and M0^mts^ patients. (**B**) The number of EpCAM-CK7/8-CD24+CD44±N-cadherin+ CCs in in M0 and M0^mts^ patients. P-values represented for all comparison groups.

It turned out that number of EpCAM-CK7/8-CD24+N-cadherin- CCs was higher in M0^mts^ patients (542.00 (220.00–2970.00) cells/mL) compared to patients without metastases in follow-up period (110.00 (27.35–304.00) cells/mL (p = 0.0019) (Fig. 7A). There were no significant differences in the number of EpCAM-CK7/8-CD24+N-cadherin+ CCs in groups of M0 and M^mts^ breast cancer patients (p > 0.005) cells/mL (Fig. 7B).

### Prognostic significance of the CD45-EpCAM-CK7/8-CD24+N-cadherin- CCs in breast cancer patients

**Figure 8:**
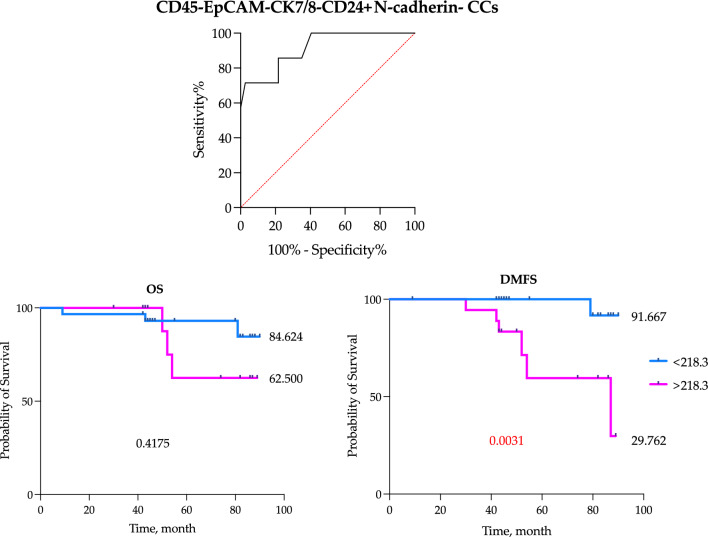
Distant metastasis-free and overall survival of breast cancer patients in relation to the number of CD45-EpCAM-CK7/8-CD24+N-cadherin- CCs.

Considering that the number of EpCAM-CK7/8-CD24+N-cadherin- CCs was increased in patients with distant metastasis during the follow-up period, we determined the cutoff point of CD45-EpCAM-CK7/8-CD24+N-cadherin- CCs count with the highest prognostic value for distant metastases in M0 breast cancer patients using ROC analysis (AUC 0.91 (0.79–1.00), p = 0.0006, sensitivity—85.7%, specificity—78.4%) (Fig. 8).

Further we assessed DMFS for breast cancer patients (M0 and M0^mts^, n = 48) with CD45-EpCAM-CK7/8-CD24+N-cadherin- CCs count below and above established cut-off (218.3 cells/1 ml). We took into consideration that significant proportion of patients from M0^mts^ group was represented by large tumor size (T4) compared to M0 group (p = 0.008). However, it turned out that the number of CD45-EpCAM-CK7/8-CD24+N-cadherin- CCs was independent of stage T and the median in patients with T1, T2, T4 was 137.4 (38.35–307) cells/mL, 120.0 (27.35–390) cells/mL and 220.0 (120.0–2982) cells/mL, respectively.

We assessed outcomes at a 90 months follow-up. In patients with CD24+N-cadherin- CCs count above the cut-off distant metastasis-free survival rate was significantly lower at 29.762%, while in patients with number of cells below the cut-off – 91.667% (HR = 12.06 (2.501–58.10), p = 0.0031) (Fig. 8). Overall survival was independent of the number of CD24+N-cadherin- CCs.

The number of CD45-EpCAM-CK7/8-CD24+N-cadherin- CCs above the cut-off was a prognostic factor of poor distant metastasis-free survival in breast cancer patients (Table 3).

**Table 3:** The univariate and multivariate Cox-regression analyses of distant metastasis-free for breast cancer patients.

Variable	Univariate			Multivariate		
	HR	95% CI	P value	HR	95% CI	P value
Cut-off > 218.3 [Yes]	13.65	2.288 to 259.7	0.0163	11.72	1.533 to 268.4	0.0431
NACT [Yes]	4.877	1.028 to 34.46	0.0611	8.675	0.2203 to 421.1	0.2422
LN [Yes]	10.35	2.070 to 76.91	0.0078	11.16	1.718 to 146.8	0.0238
Tumor size	1.606	0.6219 to 3.385	0.2590	0.4831	0.1254 to 1.661	0.2539
Age	0.9659	0.8963 to 1.034	0.3331	1.032	0.9011 to 1.195	0.6463
Unfavorable molecular type [Yes]	1.441	0.2598 to 7.059	0.6527	0.3867	0.02.076 to 4.523	0.4664

We carried out the comparison of two prognostic models using Akaike information criterion (AIC), one of which was based on established cut-off of CD45-EpCAM-CK7/8-CD24+N-cadherin- CCs count; while the second model integrated clinicopathological parameters including tumor size, molecular subtype, age, lymph node involvement and neoadjuvant chemotherapy treatment. It turned out that the first model considering the number of CD45-EpCAM-CK7/8-CD24+N-cadherin- CCs was most preferable (p = 0.0163).

To elucidate the origin of CD45-EpCAM-CK7/8-CD24+N-cadherin- CCs we conducted DNA ploidity analysis to using CopyKAT tool. We utilized scRNA-seq data generated in our previous study which are available via BioProject under the accession number PRJNA776403^17^. It was revealed that 2 out of 6 of the identified cells had an abnormal number of chromosomes, while 4 out of 6 were diploid.

## Discussion

Over the past 15 years, following the registration of CELLSEARCH technology for CTC detection, numerous alternative platforms have been conceived and introduced to the global market. Among these, systems such as CanPatrol, RareCyte and AdnaTest^18,19^ have emerged. Notably, these technologies primarily focus on the detection of CTCs expressing EpCAM and cytokeratin. Nonetheless, this has not translated into the widespread adoption of CTC detection technology in clinical practice. This seems to stem from the fact that commercially available platforms do not harness the complete predictive potential of CTC enumeration. The CTCs number detected in peripheral blood before and during treatment is an independent predictor of progression-free survival (PFS) and overall survival (OS). The favorable prognosis was observed in patients with less than 5 CTCs per 7.5 mL of peripheral blood regardless of primary tumor histology, molecular subtype, localization of first metastases, or whether the patient had recurrence of the disease^12,20^. Nevertheless, it is crucial to emphasize that there has been a no significant success in CTCs detection for predicting the risk of distant metastasis.

Probably, this may be since only a small part of CTCs is pathogenetically significant, which is confirmed by the data on the low percentage of cells with metastatic potential (0.01%) and high heterogeneity^21,22^. To date remains unclear what characteristics could distinguish metastasis-associated CTCs subpopulation from the total pool of CTCs. A definitive breakthrough was also lacking in resolving this issue by considering the stemness and EMT characteristics of CTCs.

Considerable attention has shifted toward tumor cells expressing CD24, which hold prognostic significance in breast cancer. It has been shown that increased CD24 gene expression in the primary tumor was associated with HER2-overexpression, TNBC subtype, high risk of distant metastasis, and short overall and recurrence-free survival^23–25^.

Moreover, there is data on the prognostic value of CD24 expression on CTCs in breast cancer. However, this association was not observed for the overall count of CTCs but for subpopulation with specific phenotype. Namely, CD24 positive expression in CTCs with hybrid EMT phenotype was closely associated with stage, lymph node metastasis and tumor size, but not with distant metastasis in breast cancer patients^9^.

The association between CD24 expression and a poor prognosis seems to be attributed to the role of CD24 in regulation of tumor cell migration, invasion, and proliferation^5^. In addition, CD24 expression on tumor cells in TNBC and ovarian cancer was found to disrupt immune response by acting as an antiphagocytic surface protein, which has been termed a "don't eat me" signal^25^.

Our study enabled us to investigate the potential correlation between circulating cells (CTCs, Epit-CCs and CCs) in the peripheral blood of breast cancer patients and the occurrence of distant metastases, with a specific focus on the CD24+ cells. We studied the association of CTCs and different subpopulations of CCs with distant metastasis in two groups: patients with metastases in follow-up period (M0^mts^) (median was 90 month), and in patients with metastases occurred before the study (M1). The detection of the EpCAM, CK7/8 and N-cadherin expression on studied cells allowed us to determine variants of EMT phenotypes. In 2020, group of researchers proposed a consensus for determining the molecular manifestations of EMT^26^. However, these guidelines lack clear criteria for categorizing tumor cells into distinct EMT phenotypes. Consequently, it is widely acknowledged to differentiate between epithelial, mesenchymal, and mixed (hybrid) EMT phenotypes^27,28^. Hybrid tumor cells exhibit greater plasticity and metastatic potential when compared to cells with epithelial or mesenchymal EMT phenotypes^29^.

In our study, loss of membrane expression of EpCAM (EpCAM-CK7/8+), which is often associated with nuclear translocation, as well as loss of sole CK7/8 expression (EpCAM+ CK7/8-) or co-expression of EpCAM and CK7/8 (EpCAM+CK7/8+) in the absence of N-cadherin are consistent with epithelial EMT phenotypes. N-cadherin expression in the absence of EpCAM and CK7/8 is considered a manifestation of the mesenchymal phenotype of EMT. Whereas co-expression of any epithelial marker and N-cadherin suggests a hybrid phenotype of EMT^30^.

The most significant challenges arise when attempting to identify the terminal stage of EMT. Theoretically, one of the characteristics of the terminal EMT phenotype should be the absence of expression of epithelial markers, specifically, EpCAM and CK7/8 in our study. In such case, a challenge arises in distinguishing these cells with a terminal EMT phenotype from true mesenchymal cells.

Considering that the studied cells might encompass not only CTCs displaying distinct epithelial features but also cells exhibiting a terminal EMT phenotype without the expression of EpCAM and CK7/8. That is why we initially assessed the correlation between the count of CD45-CD24+ CCs and distant metastasis. No publications regarding the clinical significance of CD45-CD24+ CCs were identified in our investigation. In our study, CD45-CD24+ CCs were associated with distant metastasis, as their count was elevated in M0^mts^ and M1 breast cancer patients compared to the M0.

The subdivision of CD45-CD24+ cells detected in the bloodstream into true CTCs, CCs with any variants of studied epithelial markers and CCs lacking their expression, yielded an unexpected outcome. First, the number of CTCs was not associated with distant metastasis in either M0^mts^ or M1 patients. This applies to both CD24− and CD24+ CTCs when assessed separately. Furthermore, the investigation of CCs expressing various combinations of EpCAM and CK7/8 failed to identify a population with prognostic significance for breast cancer metastases. Surprisingly, we found one population that was decreased in metastatic breast cancer patients (M1) compared to patients in groups with no metastasis (M0) and those with metastasis during the follow-up period (M0^mts^). Second, the count of CD45-EpCAM-CK7/8-CD24+ CCs was notably higher in both the M0^mts^ and M1 groups when compared to the M0 group, with the prognostic value depending on N-cadherin expression. In the prospective study (M0^mts^ patients) it appeared that the expression of CD44 was irrelevant for metastasis prediction, while the absence of N-cadherin expression was crucial. Therefore, CCs exhibiting the CD45-EpCAM-CK7/8-CD24+N-cadherin- phenotype demonstrated the most pronounced prognostic significance in assessing the risk of distant metastasis occurrence. Additionally, having a number of these cells above the established cutoff (218.3 cells/1 mL) predicted a threefold shorter metastasis-free survival during follow-up compared to patients whose EpCAM-CK7/8-CD24+CD44±N-cadherin- cell count was less than 218.3 cells/1 mL (HR = 12.06). However, no association with overall survival was established. Utilizing this parameter alone, the Cox regression model exhibited superior predictive capabilities compared to a model based on conventional prognostic criteria for breast cancer.

In contrast, the EpCAM-CK7/8-CD24+CD44+N-cadherin+ phenotype was associated with the M1 group of patients. This finding is intriguing due to the presumed pathogenetic involvement of these cells in metastatic disease and their potential as indicators of the effectiveness of adjuvant therapy in M1 patients.

Above, we discussed the probability of the tumor origin of CD45-EpCAM-CK7/8- CCs as cells with a terminal EMT phenotype. A high probability of the latter is indicated by the aneuploidy observed in a portion of cells with the corresponding genotype. An alternative hypothesis is to suggest that indicated may be immature cells originating from the bone marrow. Nonetheless, if the second hypothesis holds true, it's worth noting that the mentioned CCs do not align with any of the recognized stages of monocyte or neutrophil maturation, primarily due to the presence of CD45 expression in the myeloid cells^31^.

The results obtained indicate the presence of CCs in the peripheral blood of breast cancer patients, predicting the high risk of distant metastasis occurrence and characterized by an unspecified genesis, allowing both tumor and myeloid origin. This hypothesis is confirmed by presence of both cells with a normal number of chromosomes and cells with aneuploidy among cells with the corresponding genotype in breast cancer patients.

Apparently, the heterogeneous CD45-EpCAM-CK7/8-CD24+N-cadherin- CCs population includes genuinely mesenchymal cells, the origin and physiological functions of which are yet to be clarified, as well as cells that may be of tumor origin with a preterminal EMT phenotype.

It remains unclear, whether the identified subpopulation play a pathogenetic role in the distant metastases formation as "seeds" or contribute to the premetastatic niches establishment, or whether they serve only as valuable indicators of the metastatic process.

## Conclusion

Utilizing a panel of antibodies against EpCAM, CK7/8, CD24, CD44, and N-cadherin enabled the concurrent detection of EpCAM+CK7/8+ CTCs and circulating cells with varying co-expression patterns of epithelial markers (EpCAM and CK7/8), as well as CCs in which EpCAM and CK7/8 expression was not observed. In our study, CTCs and CCs exhibiting different epithelial phenotypes did not show a significant association with either the occurrence of distant metastases or distant metastasis-free survival. In contrast to CTCs, the number of CCs with CD45-EpCAM-CK7/8-CD24+N-cadherin- phenotype was associated with distant metastasis, as their numbers were elevated in patients with metastases in follow-up period (M0^mts^) compared to patients without metastases in follow-up period (M0). The presence of cells with aneuploidy among the identified CCs suggests that some of these cells originate from tumors.

The study was supported by the Russian Science Foundation (Grant # 23-15-00135).

### Supplementary Information

Supplementary Information.

## Data Availability

The datasets used and/or analyzed during the current study available from the corresponding author on reasonable request.
