# Supplementary material for: EpCAM-CD24+ circulating cells associated with poor prognosis in breast cancer patients
Source: Sci Rep. 2024 May 28;14:12245. doi: 10.1038/s41598-024-61516-2 (PMC11133449; doi:10.1038/s41598-024-61516-2)
Supplement: Supplementary file 1 — Supplementary Information. [file 41598_2024_61516_MOESM1_ESM.pdf]

Supplementary

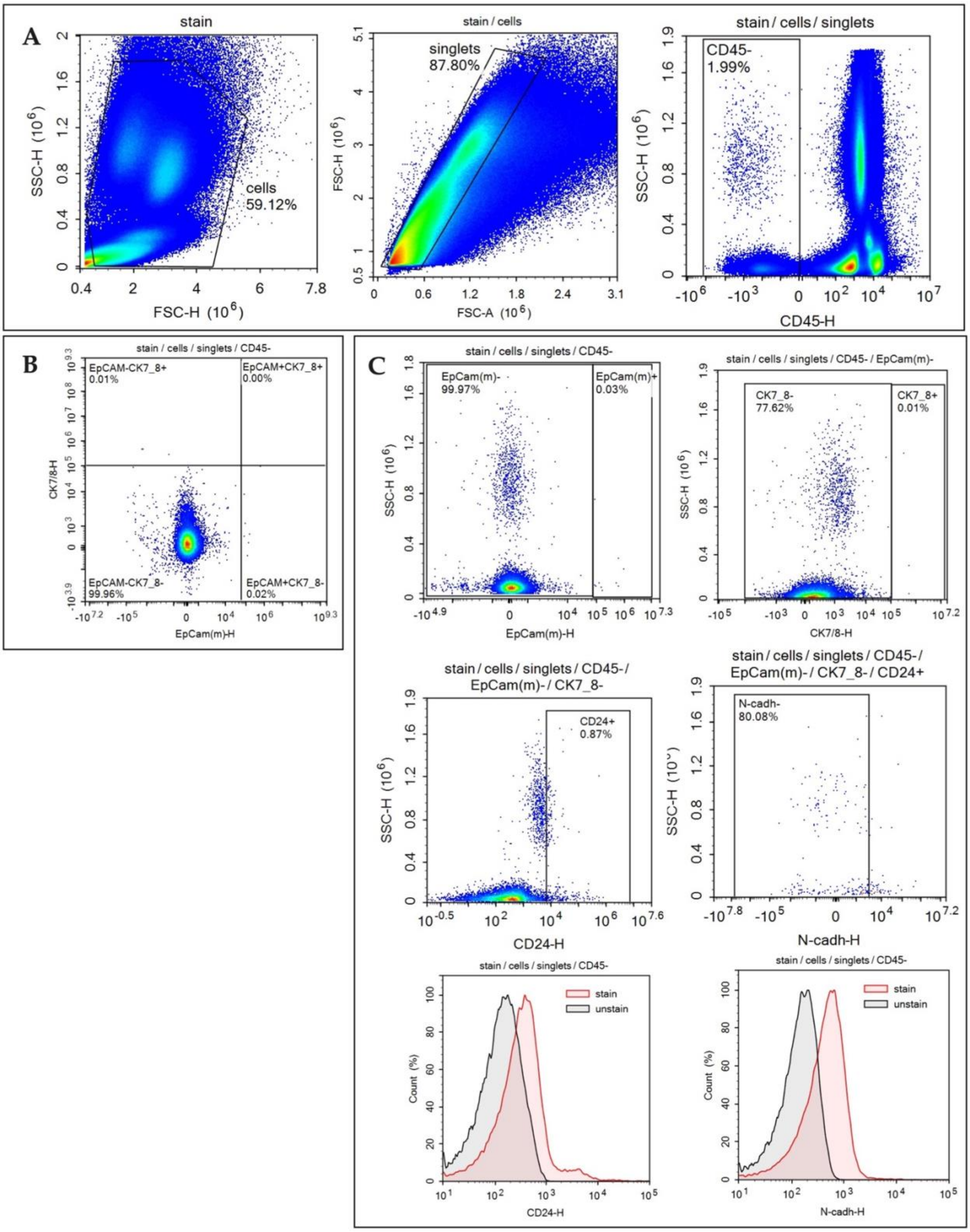

Figure 1. Flow cytometry analysis of CTCs and CCs of breast cancer patient. **A**. Gating strategy for detection of CD45-cells. **B.** Plot represents three studied CTCs subsets: EpCam+CK7/8-; EpCam-CK7/8+; EpCam+CK7/8+. **C.** Gating strategy for detection of CD45-EpCAM-CK7/8-CD24+N-cadherin- CCs.
